# Epigenetic silencing of *miR-335* and its host gene *MEST* in hepatocellular carcinoma

**DOI:** 10.3892/ijo.2012.1724

**Published:** 2012-11-30

**Authors:** OSAMU DOHI, KOHICHIROH YASUI, YASUYUKI GEN, HISASHI TAKADA, MIO ENDO, KAZUHIRO TSUJI, CHIKA KONISHI, NOBUHISA YAMADA, HIRONORI MITSUYOSHI, NOBUAKI YAGI, YUJI NAITO, SHINJI TANAKA, SHIGEKI ARII, TOSHIKAZU YOSHIKAWA

**Affiliations:** 1 Department of Molecular Gastroenterology and Hepatology, Kyoto Prefectural University of Medicine, Kyoto;; 2 Department of Hepato-Biliary-Pancreatic Surgery, Tokyo Medical and Dental University, Tokyo, Japan

**Keywords:** *miR-335*, *MEST*, methylation, hepatocellular carcinoma

## Abstract

MicroRNAs (miRNAs) are small non-coding RNAs that function as endogenous silencers of target genes. Some tumor-suppressive miRNAs are known to be epigenetically silenced by promoter DNA methylation in cancer. In the present study, we aimed to identify miRNA genes that are silenced by DNA hypermethylation in hepatocellular carcinoma (HCC). We screened for miRNA genes with promoter DNA hypermethylation using a genome-wide methylation microarray analysis in HCC cells. It was found that *miR-335*, which is harbored within an intron of its protein-coding host gene, *MEST*, was downregulated by aberrant promoter hypermethylation via further methylation assays, including methylation-specific PCR, combined bisulfite and restriction analysis, bisulfite sequencing analysis and 5-aza-2′-deoxycytidine treatment. The expression levels of *miR-335* significantly correlated with those of *MEST*, supporting the notion that the intronic *miR-335* is co-expressed with its host gene. The levels of *miR-335*/*MEST* methylation were significantly higher in 18 (90%) out of 20 primary HCC tumors, compared to their non-tumor tissue counterparts (P<0.001). The expression levels of *miR-335* were significantly lower in 25 (78%) out of 32 primary HCC tumors, compared to their non-tumor tissue counterparts (P=0.001). Furthermore, the expression levels of *miR-335* were significantly lower in HCC tumors with distant metastasis compared to those without distant metastasis (P=0.02). In conclusion, our results indicate that expression of *miR-335* is reduced by aberrant DNA methylation in HCC.

## Introduction

Hepatocellular carcinoma (HCC) is the fifth most common malignancy in men and the eighth most common in women worldwide. It is estimated to cause approximately half a million deaths annually (1). Several risk factors for HCC have been reported, including infection with hepatitis B and C viruses, dietary intake of afratoxin and alcohol consumption. However, the molecular pathogenesis of HCC remains poorly understood.

MicroRNAs (miRNAs) are ∼22 nucleotide non-coding RNAs that function as endogenous silencers of target genes. Currently, more than 1,000 miRNAs have been identified in the human genome (the miRBase database) and each miRNA is predicted to control hundreds of gene targets. miRNAs are expressed in a tissue-specific manner and play important roles in development, cell proliferation, apoptosis and oncogenesis (2–5). Dysregulation of miRNAs in cancer has been repeatedly described (6–8). HCC is no exception and various HCC-specific miRNA signatures have been described (9).

DNA methylation of CpG islands within the promoter regions of tumor suppressor genes is known to inhibit transcriptional initiation and thereby silence these genes. Growing evidence indicates that some tumor-suppressive miRNAs are also epigenetically silenced by promoter DNA methylation in cancer (10), suggesting the diagnostic and therapeutic potential of these miRNAs.

In the present study, we aimed to identify miRNA genes that are silenced by DNA hypermethylation in HCC. We screened for genes with promoter DNA hypermethylation using a genome-wide methylation microarray analysis and found that *miR-335*, which is harbored within an intron of its protein-coding host gene *MEST*, is downregulated by aberrant promoter hypermethylation in HCC.

## Materials and methods

### Cell lines and primary tumors

The following 21 HCC cell lines were examined: HLE, HLF, PLC/PRF/5, Li7, Huh7, Hep3B, SNU354, SNU368, SNU387, SNU398, SNU423, SNU449, SNU475, JHH-1, JHH-2, JHH-4, JHH-5, JHH-6, JHH-7, Huh1 and HepG2 (11).

Paired tumor and non-tumor tissues were obtained from 32 HCC patients who underwent surgery. All specimens were immediately frozen in liquid nitrogen and were stored at −80°C until further use. Genomic DNA and total RNA were isolated using the Puregene DNA isolation kit (Gentra, Minneapolis, MN) and TRIzol reagent (Invitrogen, Carlsbad, CA), respectively. Twenty tumor samples were available for DNA methylation analyses and 32 paired tumor and non-tumor samples were available for microRNA and mRNA analyses. This study was approved by the ethics committees and conducted in accordance with the Declaration of Helsinki. Informed consent was obtained from each patient.

### Methylation array analysis

We performed a genome-wide DNA methylation analysis called microarray-based integrated analysis of methylation by isoschizomers (MIAMI), as previously described (12–14). The complete experimental procedure can be obtained at http://grc.dept.med.gunma-u.ac.jp/~gene/image/MIAMI%20Protocol%20V4.pdf. Changes in methylation were judged by assessing the differences in methylation-sensitive *Hpa*II cleavage and methylation-insensitive *Msp*I cleavage between samples. We used a custom microarray, which contains ∼38,000 probes chosen from the Agilent promoter array, on an eArray system (http://earray.chem.agilent.com/earray/).

### TaqMan miRNA assay

Reverse transcription (RT) reactions and real-time quantitative polymerase chain reactions (PCR) were performed using the TaqMan MicroRNA RT kit (Applied Biosystems, Darmstadt, Germany), TaqMan MicroRNA Assays (Applied BioSystems) and ABI PRISM 7300 Fast Real-time PCR system (Applied Biosystems), according to the manufacturer’s instructions. *RNU6B* was used as an endogenous control for miRNA levels.

### Drug treatment

Cells were treated with 1 or 5 *μ*M of 5-aza-2′-deoxycytidine (5-aza-dCyd; Sigma-Aldrich, St. Louis, MO) for 4 days or 50 ng/ml of trichostatin A (TSA; Wako, Osaka, Japan) for 1 day. In assessing drug synergy, cells were cultured in the presence of 1 or 5 *μ*M of 5-aza-dCyd for 4 days and were then treated for an additional 24 h with 50 ng/ml of TSA.

### Methylation analysis

Methylation status was examined by methylation-specific PCR (MSP), bisulfite PCR followed by restriction enzyme digestion [combined bisulfite and restriction analysis (COBRA)] (15) and bisulfite sequencing analysis, as previously described (16). The primers used are listed in Table I. Briefly, for MSP, genomic DNA was treated with sodium bisulfite using an EZ DNA Methylation kit (Zymo Research, Orange, CA) and subjected to PCR using specific primer sets. For COBRA, genomic DNA was treated with sodium bisulfite and subjected to PCR. The PCR products were digested with *Bst*UI, which recognizes sequences unique to the methylated alleles, but cannot recognize unmethylated alleles and the digested products were electrophoresed on 3% agarose gels and stained with ethidium bromide. Methylation levels were calculated as the ratio of the gray scale value of the methylated band to that of the combined methylated and unmethylated bands. The gray scale value was obtained by scanning the gel with Adobe Photoshop CS3 Extended software (Adobe Systems Inc., San Jose, CA, USA). For bisulfite-sequencing, the PCR products were cloned and then sequenced. CpGenome universal unmethylated and methylated DNA (Chemicon, Billerica, MA) served as controls for unmethylated and methylated DNA, respectively.

### Real-time quantitative RT-PCR

We quantified mRNA using a real-time fluorescence detection method, as previously described (11). Real-time quantitative PCR experiments were performed with the LightCycler system using FastStart DNA Master Plus SYBR Green I (Roche Diagnostics, Penzberg, Germany), according to the manufacturer’s protocol. The primers used are listed in Table I. The endogenous control for mRNA was *GAPDH*.

### Statistical analysis

Spearman’s rank correlation test, Wilcoxon signed-rank test and Mann-Whitney U test were performed using SPSS 15.0 software (SPSS, Inc., Chicago, IL). P-values of <0.05 were considered significant.

## Results

### Genome-wide DNA methylation profiles in HCC

**Figure 1.**
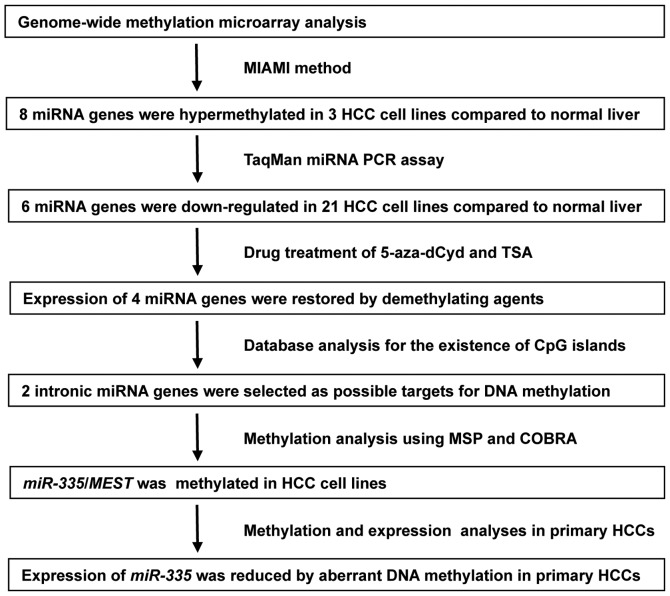
Schematic strategy for the identification of epigenetically silenced miRNA genes in HCC.

To identify miRNA genes that are silenced by DNA hypermethylation in HCC, we compared DNA methylation profiles between three HCC cell lines (SNU449, Li-7 and PLC/PRF/5) and one normal liver tissue using the MIAMI method. The microarray covers approximately 38,000 probes (corresponding to promoter regions of about 14,000 genes), which include 411 probes for miRNA (167 miRNA genes). MIAMI analyses revealed that 575 probes (484 genes) were hypermethylated and 350 probes (277 genes) were hypomethylated similarly in the three HCC cell lines compared to normal liver. The hypermethylated genes included eight miRNA genes (*miR-let-7b, miR-101-2, miR-122a, miR-146b, miR-149, miR-200b, miR-335* and *miR-497*). Therefore, further analysis was focused on these eight miRNA genes. The strategy and partial results are shown in Fig. 1.

### Expression of candidate miRNAs in HCC cell lines

**Figure 2.**
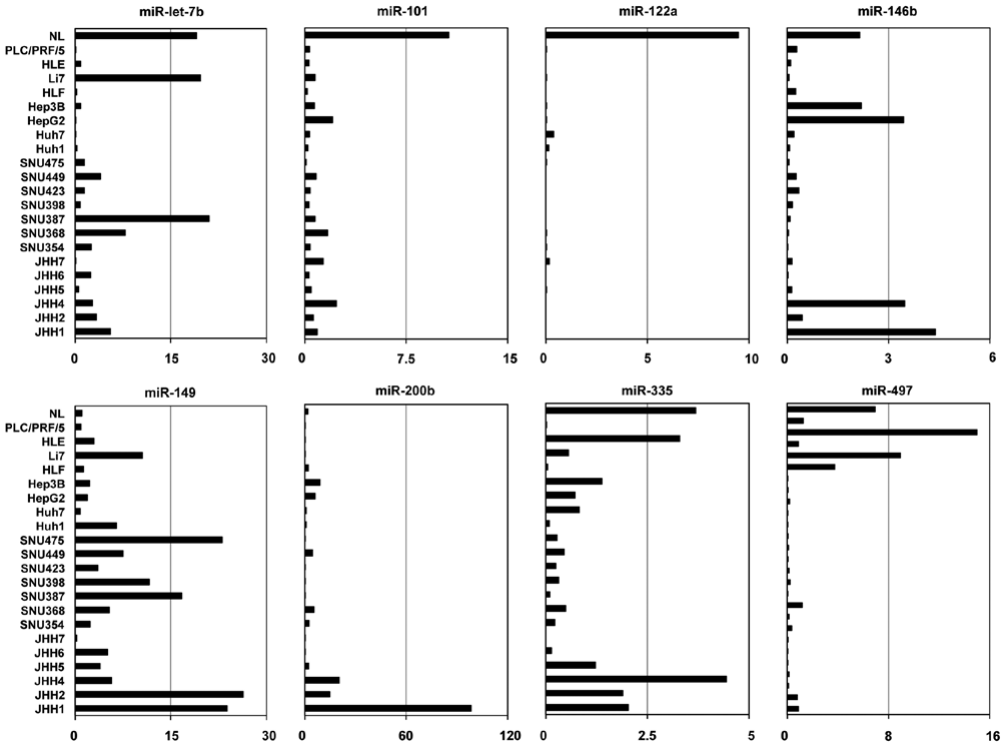
Relative expression levels of eight candidate miRNA genes, as evaluated by TaqMan miRNA PCR in 21 HCC cell lines and normal liver (NL).

We analyzed the expression levels of the eight miRNAs in 21 human HCC cell lines and normal liver using TaqMan miRNA PCR. Expression levels of six miRNAs (*miR-let-7b, miR-101-2, miR-122a, miR-146b, miR-335* and *miR-497*), but not two of the miRNAs (*miR-149* and *miR-200b*), were lower in more than half of the 21 cell lines than normal liver (Fig. 2).

### Restoration of miRNA expression by the methyltransferase inhibitor

**Figure 3.**
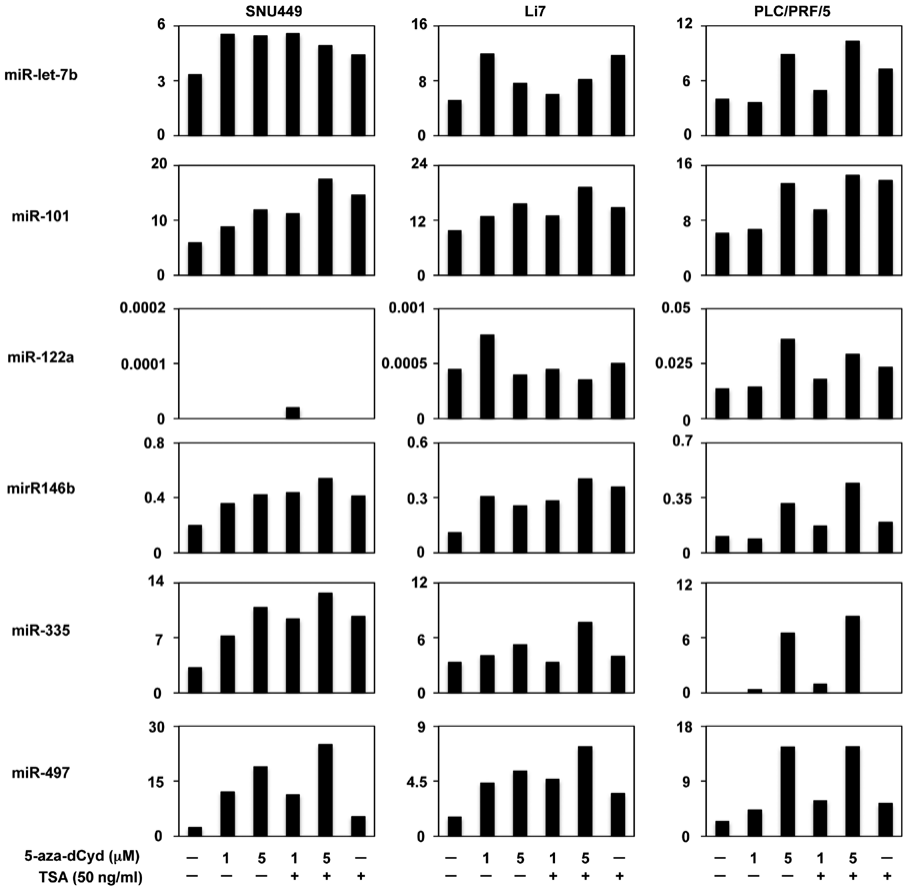
Effects of 5-aza-dCyd and TSA treatment on the expression of six candidate miRNA genes. Expression levels of six miRNA genes were determined via TaqMan miRNA PCR in SNU449, Li7 and PLC/PRF/5 cells with or without treatment with 5-aza-dCyd (1 or 5 *μ*M) for 4 days and/or TSA (50 ng/ml) for 24 h.

We then assessed the effects of demethylation on the expression of the six candidate miRNAs. Three HCC cell lines (SNU449, Li7 and PLC/PRF/5) were treated with 5-azadCyd, a methyltransferase inhibitor and miRNA expression levels were assayed with TaqMan miRNA PCR. Expression of four miRNAs (*miR-101-2, miR-146b, miR-335* and *miR-497*), but not two of the miRNAs (*miR-let-7b* and *miR-122a*), were restored with 5-aza-dCyd treatment in all three HCC cells (Fig. 3), suggesting that aberrant DNA methylation suppressed the expression of these four miRNAs. Additionally, it was observed that treatment with a histone deacetylase inhibitor, TSA, enhanced the expression of these four miRNAs by 5-aza-dCyd in all three cell lines (Fig. 3). These findings suggest that histone deacetylation may also contribute to the transcriptional repression of these four miRNAs.

### Methylation of miR-335/MEST in HCC cells

**Figure 4.**
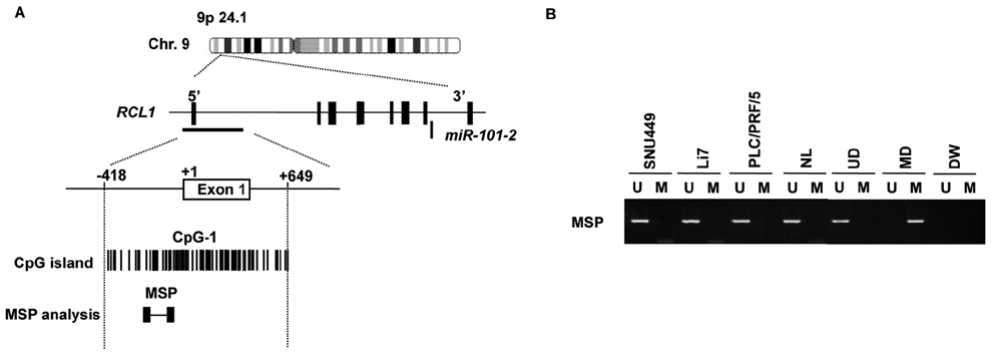
Analysis of *miR-101-2/RCL1* methylation. (A) Schematic map of the CpG island extending into exon 1 of *RCL1*. Exon 1 is indicated by an open box and the transcription start site is marked at +1. CpG sites are indicated by the vertical ticks. The region selected for MSP is indicated. (B) MSP analysis of *miR-101-2/RCL1* in the three indicated HCC cell lines (SNU449, Li7 and PLC/PRF/5) and in normal liver cells (NL). Parallel amplification reactions were performed using primers specific for unmethylated (U) or methylated (M) DNA. Unmethylated DNA (UD) and methylated DNA (MD) were used as controls. DW is a deionized water control.

**Figure 5.**
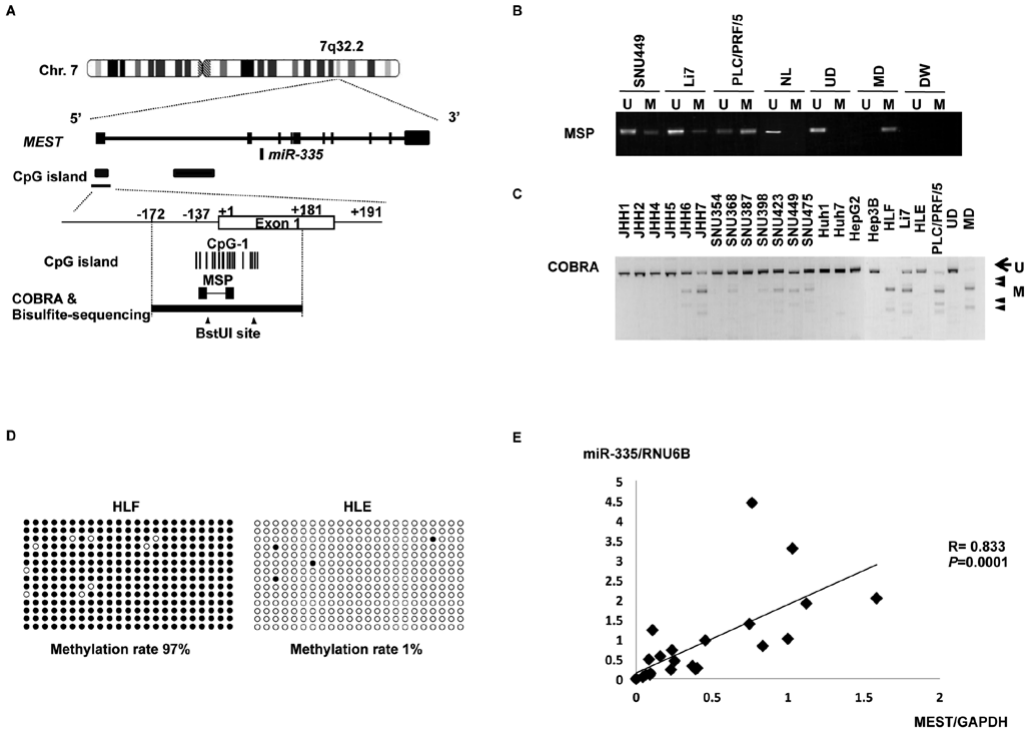
Analysis of *miR-335/MEST* methylation. (A) Schematic map of the CpG island extending into exon 1 of *MEST*. Exon 1 is indicated by an open box and the transcription start site is marked at +1. CpG sites are indicated by vertical ticks. The regions selected for MSP, COBRA and bisulfite-sequencing are indicated. The restriction site for *Bst*UI is indicated by the black arrowhead. (B) MSP analysis of *miR-335/MEST* in the three indicated HCC cell lines (SNU449, Li7 and PLC/PRF/5) and normal liver (NL). Parallel amplification reactions were performed using primers specific for unmethylated (U) or methylated (M) DNA. Unmethylated DNA (UD) and methylated DNA (MD) were used as controls. DW is a deionized water control. The three cell types yielded both methylated and unmethylated products, whereas normal liver displayed exclusively unmethylated products. (C) COBRA of *miR-335/MEST* in the 21 HCC cell lines. The arrow and arrowheads indicate undigested products (U, unmethylated DNA) and digested fragments (M, methylated DNA), respectively. (D) Bisulfite-sequencing of two HCC cell lines (HLF and HLE). All 23 CpG sites were sequenced. Each circle indicates unmethylated (open circles) and methylated (solid circles) CpG dinucleotides. Percentages indicate the fraction of methylated CpG dinucleotides. (E) Correlation between the expression levels of *miR-335* and *MEST* in 21 HCC cell lines.

About half of all miRNA genes are encoded in the introns of protein-encoding genes and subsequently excised from a primary transcript in common with protein coding genes, so-called host genes (10,17–19). Thus, these miRNA genes are more likely to be susceptible to transcriptional repression by aberrant DNA methylation of CpG islands located in the host genes. Of the selected four genes (*miR-101-2, miR-146b, miR-335* and *miR-497*), we identified that *miR-101-2* and *miR-335* are intronic miRNAs using the human genome browser at UCSC (February 2009). *miR-101-2* and *miR-335* are located within the introns of RNA terminal phosphate cyclase-like 1 gene (*RCL1)* (Fig. 4A) and mesoderm specific transcript homolog gene (*MEST*) (Fig. 5A), respectively. We also found CpG islands around the transcription start sites of m*iR-101-2/RCL1* and *miR-335*/*MEST* genes using the genome database of the European Bioinformatics Institute. However, no CpG islands were found around *miR-146b* or *miR-497*.

Therefore, we assessed the methylation status of the CpG islands of *miR-101-2/RCL1* and *miR-335/MEST* via MSP in three HCC cells (SNU449, Li7 and PLC/PRF/5) and normal liver. MSP analyses indicated that the CpG island of *miR-101-2/RCL1* was not methylated in these HCC cells (Fig. 4B), whereas aberrant DNA methylation within the CpG island of *miR-335*/*MEST* was evident in all three HCC cells (Fig. 5B).

To confirm and quantify the methylation status of *miR-335/MEST*, we assayed DNA methylation levels of the *miR-335/MEST* CpG island using the COBRA technique, which involves bisulfite PCR followed by restriction enzyme digestion, in 21 HCC cell lines. COBRA analyses (Fig. 5C) revealed that the *miR-335*/*MEST* CpG island was hypermethylated in three cell types (JHH7, HLF and PLC/PRF/5) that lack the expression of *miR355* (Fig. 2), partly methylated in eight (JHH6, SNU368, SNU398, SNU423, SNU449, SNU475, Huh7 and Li7) with reduced expression of *miR355* (Fig. 2) and unmethylated in the remaining 10 cell lines, including HLE. Consistent with the results of COBRA, further analysis of the PCR products with bisulfite-sequencing showed that the CpG island was hypermethylated in HLF cells (methylation rate, 97%) and hypomethylated in HLE cells (methylation rate, 1%) (Fig. 5D). Taken together, these data suggest that the *miR-335*/*MEST* CpG island was hypermethylated in some HCC cells. The physical relationship between *miR-335*, *MEST*, the CpG island and the primers used for MSP and COBRA are shown in Fig. 5A.

The expression levels of *miR-335* significantly correlated with those of *MEST* in 21 HCC cell lines (Spearman’s rank correlation test, r=0.83; P=0.0001) (Fig. 5E), supporting the notion that the intronic *miR-335* is co-expressed with its host gene, *MEST*, under the control of the host gene promoter.

### Methylation and reduced expression of miR-335 in primary HCC tumors

**Figure 6.**
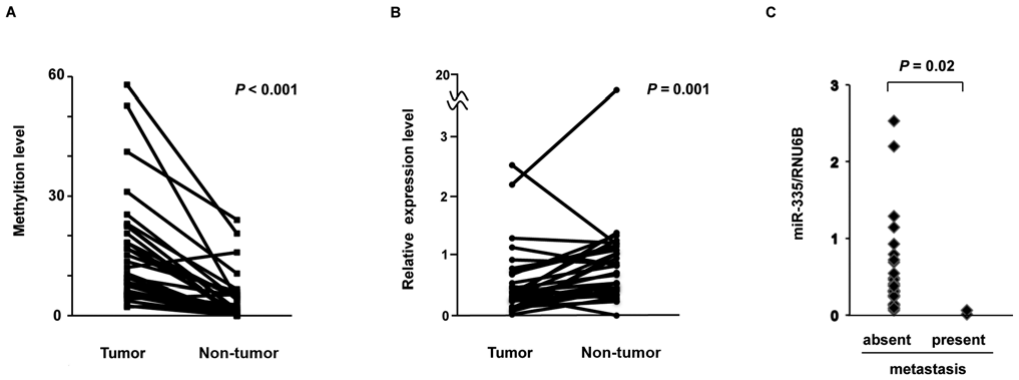
Methylation and reduced expression of *miR-335* in primary HCC tumors. (A) Plot of the methylation levels of *miR-335/MEST* in paired tumors and non-tumor tissues from 20 patients with primary HCCs. Methylation levels were determined by COBRA, as described in Materials and methods and were expressed as a percentage of the methylated DNA positive control value. The value obtained for the unmethylated DNA control was used as the baseline (0%). (B) Relative expression of *miR-335* in paired tumor and non-tumor tissues from 32 patients with primary HCC. (C) Relative expression of *miR-335* in tumors from the 32 HCC patients with or without distant metastasis.

To determine whether the methylation of the *miR-335/MEST* CpG island observed in HCC cell lines also occurs in primary human HCC, we assessed the methylation status of *miR-335/MEST* in paired tumor and non-tumor tissues from 20 patients with primary HCC by using COBRA. Methylation of *miR-335*/*MEST* was observed in all 20 HCC tumors and in 15 of the 20 non-tumor liver tissues. Although methylation of *miR-335*/*MEST* was found in both HCC tumors and non-tumor tissues, the level of *miR-335*/*MEST* methylation was significantly higher in 18 (90%) out of 20 tumors, compared to their non-tumor tissue counterparts (Wilcoxon signed-rank test, P<0.001) (Fig. 6A).

To investigate whether the reduced expression of *miR-335* observed in HCC cells was relevant in primary HCC tumors, we analyzed the expression of *miR-335* in paired tumor and non-tumor tissues from 32 HCC patients via TaqMan miRNA PCR. The expression level of *miR-335* was significantly lower in 25 (78%) out of 32 tumors, compared to their non-tumor tissue counterparts (Wilcoxon signed-rank test, P= 0.001) (Fig. 6B). Taken together, these findings suggest that the expression of *miR-335* was frequently reduced by aberrant DNA methylation in primary HCCs.

Since *miR-335* was identified as a metastasis suppressor miRNA in breast cancer by Tavazoie *et al*(20), we examined the relationship between the expression levels of *miR-335* and the presence of distant metastasis in these 32 primary HCCs. The expression of *miR-335* was significantly lower in HCC tumors with distant metastasis than in those without distant metastasis (Mann-Whitney U test, P=0.02) (Fig. 6C), suggesting that a reduced expression of *miR-335* may be association with distant metastasis in HCC, as well as in breast cancer.

## Discussion

This is the first report that *miR-335* is downregulated in HCC via aberrant promoter hypermethylation, which was demonstrated through a number of approaches. First, we screened for genes with promoter DNA methylation in HCC cell lines using MIAMI, a powerful method for genome-wide profiling of promoter methylation in the human genome (12–14) and found eight miRNA genes that were possibly methylated in HCC cells. Further methylation analyses, including MSP, COBRA, bisulfite-sequencing and drug treatment with 5-aza-dCyd and TSA, combined with expression analyses, narrowed down the candidate methylated miRNA genes and confirmed that the *miR-335*/*MEST* CpG island was hypermethylated in some HCC cells. In primary HCCs, the level of *miR-335*/*MEST* methylation was significantly higher and the expression of *miR-335* was significantly lower in tumors compared to their non-tumor tissue counterparts, suggesting that the expression of *miR-335* was reduced by aberrant DNA methylation in primary HCCs. Furthermore, our results suggest that a reduced expression of *miR-335* may be associated with distant metastasis in HCC.

DNA hypermethylation of CpG islands within promoter regions is known to be an epigenetic aberration leading to the inactivation of tumor-suppressive miRNA in cancer, which is similar to that of many classical tumor-suppressor genes. To date, 19 intergenic miRNA genes, which are located in the non-coding regions between genes and 42 intronic (intragenic) miRNA genes, which are harbored within introns of their protein-coding host genes, have been identified as tumor-suppressive miRNA (10). Of these, *miR-335* was reported to suppress metastasis and migration by targeting SOX4 and tenascin C and inhibit tumor initiation in breast cancer (20,21). The transcription of *miR-335* was shown to be co-regulated with *MEST* by promoter hypermethylation in breast cancer cells (21). Furthermore, it was demonstrated that *miR-335* regulates Rb1 and controls cell proliferation in a p53-dependent manner (22). Recent studies have shown that *miR-335* orchestrates cell proliferation, migration and differentiation in human mesenchymal stem cells (23), as well as inhibits growth and invasion of malignant astrocytoma cells (24). Further work will be aimed at elucidating the role of *miR-335* in the carcinogenesis and metastasis of HCC.

## Figures and Tables

**Table I.** Sequences of PCR primers used in the study.

Purpose	Gene	Forward primer		Reverse primer
Methylation specific primer	*miR-335/MEST*	Methylation specific primer	5′-TTGTAATAGGTGGCGTTGAC-3′	5′-ACTCGAAACTAAAACGTCGC-3′
		Unmethylation specific primer	5′-TTTTTGTAATAGGTGGTGTTGAT-3′	5′-ACTCAAAACTAAAACATCACCAA-3′
	*miR-101-2/RCL1*	Methylation specific PCR	5′-GATTGGTAATTTTCGCGTC-3′	5′-GCGCTACCATTAATCCGTA-3′
		Unmethylation specific primer	5′-GATTGGTAATTTTTGTGTT-3′	5′-ACACTACCATTAATCCATA-3′
Real-time quantitative RT-PCR	*MEST*		5′-CGCAGGATCAACCTTCTTTC-3′	5′-CATCAGTCGTGTGAGGATGG-3′
